# Gardner syndrome with odontogenic sinusitis: A case report

**DOI:** 10.1002/ccr3.4256

**Published:** 2021-06-23

**Authors:** Kosuke Saito, Motoki Sekine, Fumiyuki Goto, Hikaru Yamamoto, Shoji Kaneda, Akihiro Sakai, Hiroaki Iijima, Mayu Yamauchi, Aritomo Yamazaki, Kenji Okami

**Affiliations:** ^1^ Department of Otolaryngology‐Head and Neck Surgery Tokai University School of Medicine Kanagawa Japan

**Keywords:** familial adenomatous polyposis, Gardner syndrome, odontoma, osteoma, sinusitis

## Abstract

Gardner syndrome with odontogenic sinusitis is rare but should be suspected in patients with multiple osteomas of the skull and facial bones, excess teeth, impacted teeth, and odontomas. Early diagnosis and treatment of GS may improve prognosis.

## Graphical abstract

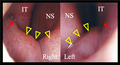

## 1 INTRODUCTION

Gardner syndrome (GS) is an autosomal dominant genetic disease and is a subtype of familial adenomatous polyposis (FAP) manifesting as soft tissue tumors and osteomas.^1^ If untreated, it can lead to colorectal cancer; hence, early diagnosis is important. Gardner syndrome with sinusitis is rare. We report a case of GS with odontogenic sinusitis.

## 2 CASE REPORT

**FIGURE 1:**
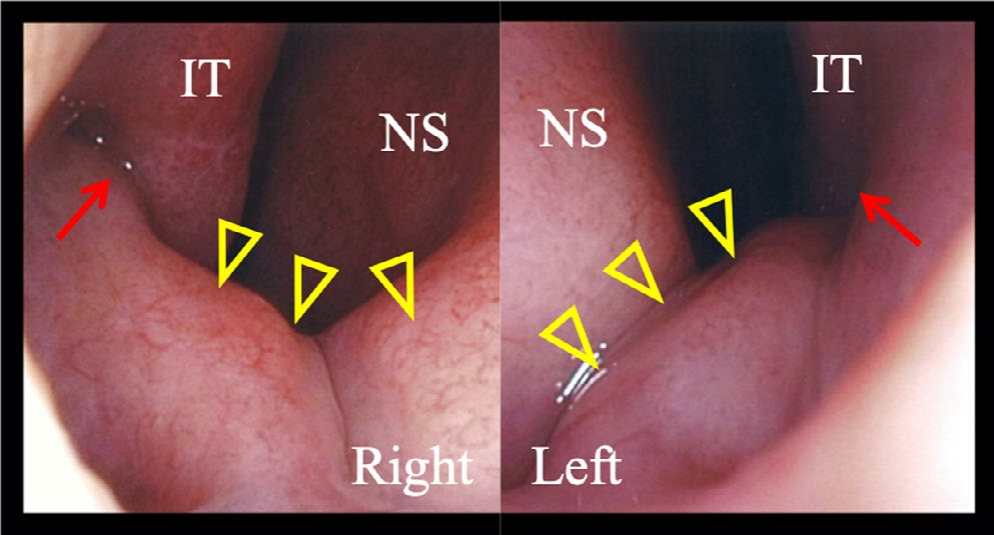
Initial endoscopic examination image shows an elevated anterior nasal floor (yellow triangles) and a closed inferior meatus (red arrows) bilaterally. (IT, inferior turbinate; NS, nasal septum)

A 29‐year‐old male presented with a 1‐month history of pain in the left cheek and a 2‐week history of swelling on the left cheek. He had a history of diabetes and was on hypoglycemic medication. His mother had died of gastric cancer, and his aunt had a history of total colectomy. An endoscopic examination revealed an elevated anterior nasal floor and closed inferior meatus bilaterally (Figure 1). Mucopurulent discharge was noted in the left middle meatus.

**FIGURE 2:**
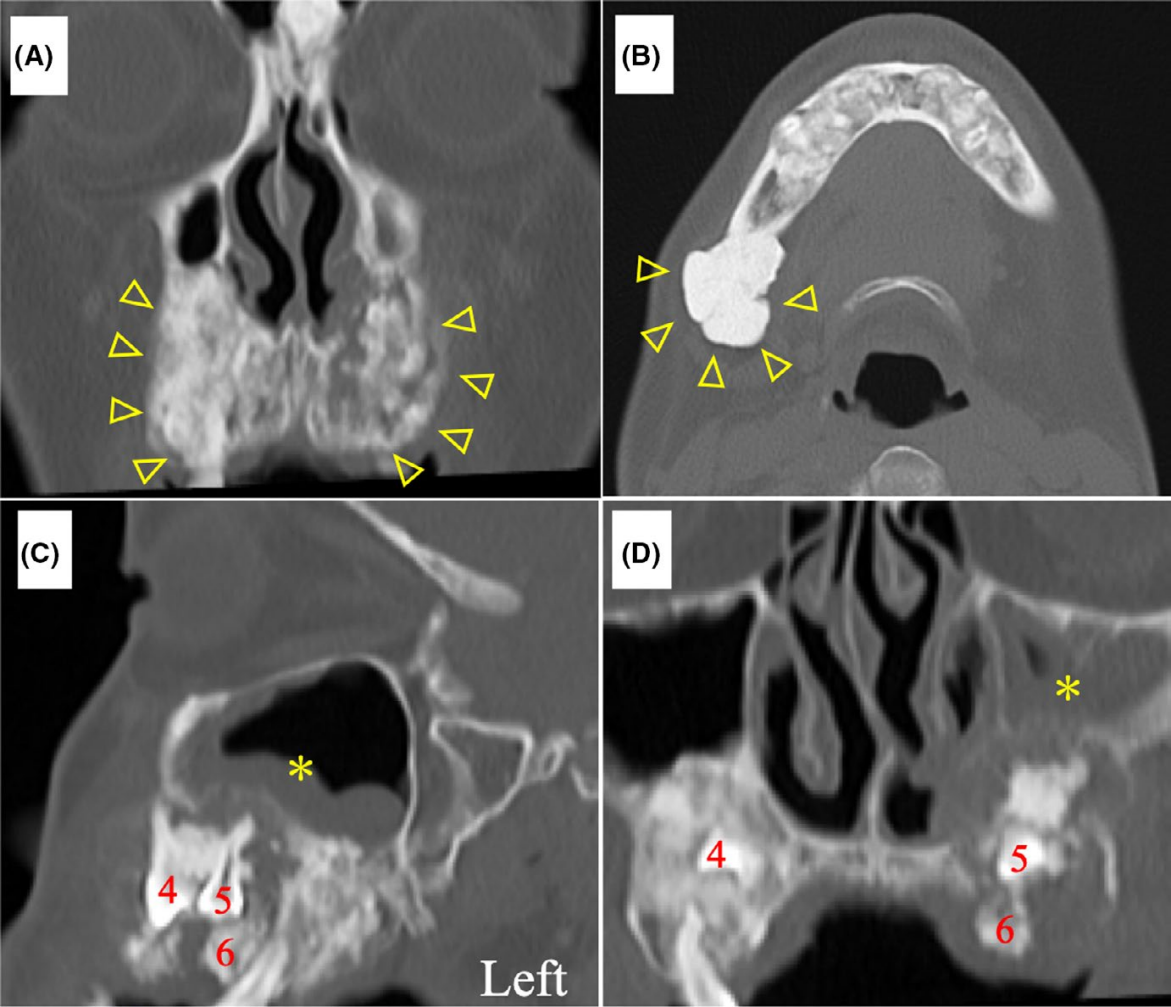
Initial computed tomography image shows nodular osteosclerosis of the maxilla and mandible (A, B; yellow triangles), multiple impacted maxillary teeth (C, D; 4, 5, and 6), soft tissue shadows (*), and bone erosion around the palatal root of the left upper first molar and upper left impacted tooth, which formed a compound odontoma. The soft tissue shadow (*) around the compound odontoma is continuous with the left maxillary sinus (C, D). (4: first premolar, 5: second premolar, 6: first molar)

**FIGURE 3:**
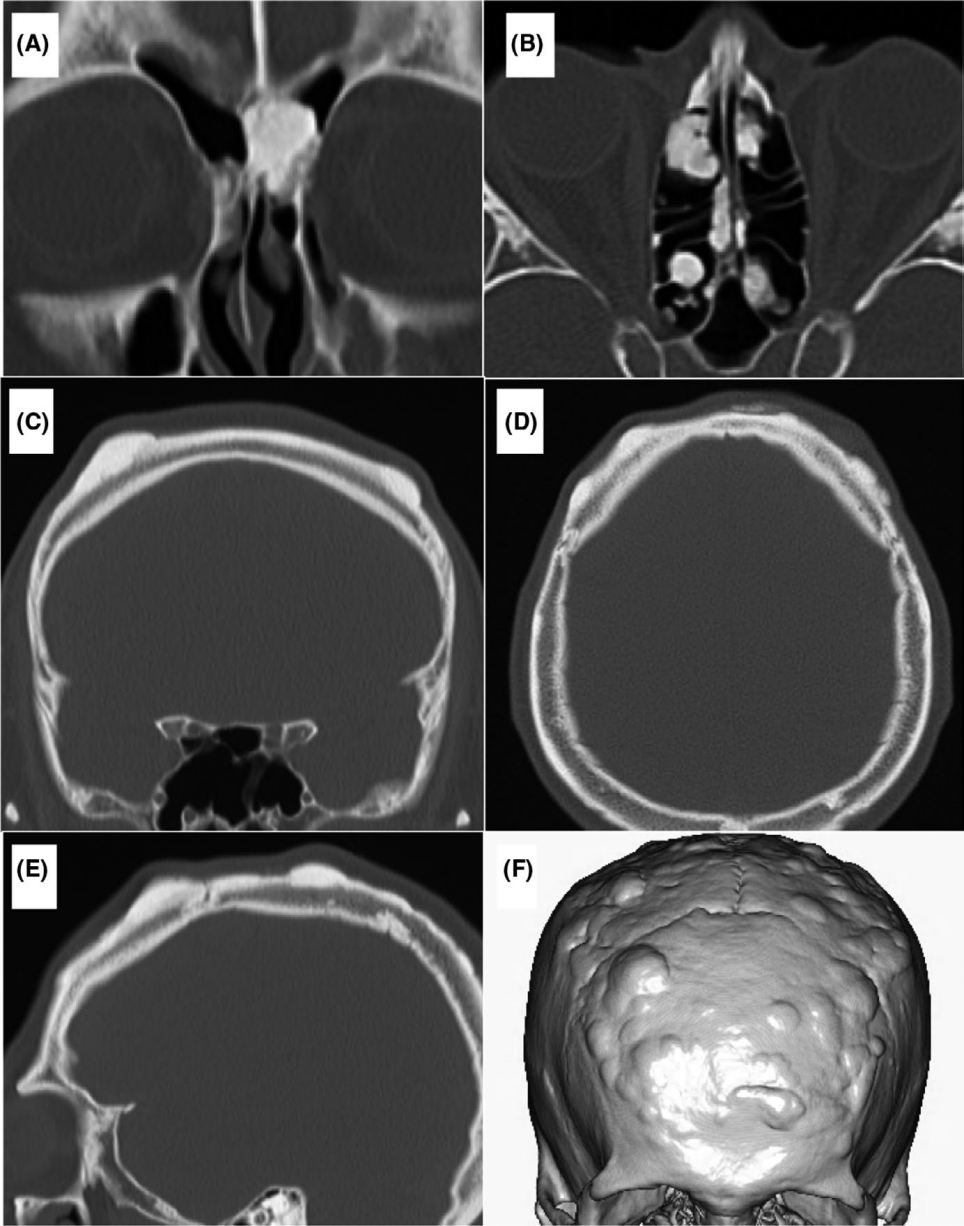
Initial computed tomography image shows multiple osteomas in the frontal sinus (A), ethmoid sinus (B), and skull (C, D, E, F)

Intraoral examination revealed multiple caries and tooth defects. Computed tomography showed nodular osteosclerosis of the maxilla and mandible (Figure 2A,B) and multiple impacted maxillary teeth (Figure 2C,D). Two upper left maxillary impacted teeth (first premolar and second premolar) formed a compound odontoma (Figure 2C). Soft tissue shadows and bone erosion were observed around the palatal root of the left upper first molar and compound odontoma. Additionally, the soft tissue shadow around the compound odontoma was continuous with the left maxillary sinus (Figure 2C,D). Multiple osteomas were found in the ethmoid sinus, frontal sinus, and skull (Figure 3). The patient was diagnosed as having left odontogenic maxillary sinusitis.

Gardner syndrome was suspected due to the positive family history, multiple osteomas in the skull and facial bone, and abnormal teeth. Multiple adenomas were found in the stomach and colon on gastrointestinal endoscopy. Furthermore, genetic testing revealed a mutation in the adenomatous polyposis coli (*APC*) gene, leading to a diagnosis of GS. Sinusitis improved following the extraction of the left upper first molar and macrolide therapy for 3 months. Prophylactic colectomy for FAP and excision surgery for odontoma have been planned.

## 3 DISCUSSION

We encountered a case of Gardner syndrome with odontogenic sinusitis. Many patients with GS are asymptomatic, and diagnosis is based on incidental findings or family history. Patients with GS might exhibit dental abnormalities such as excess teeth, impacted teeth (delayed tooth eruption), and odontoma, in addition to osteoma.^2^ Odontoma is usually asymptomatic; however, reports of infected odontoma exist.^3^ GS with odontogenic sinusitis is rare. There are no similar case reports, to the best of our knowledge. In our patient, two upper left maxillary teeth formed an odontoma, which got infected from the periapical infection of the first molar, leading to odontogenic sinusitis.

Osteoma occurs in 46%–93% of patients with GS, which is 4‐20 times more frequent than in the general population. It most commonly occurs at the outer cortex of the skull, mandible, and frontal sinus.^2^ While a single osteoma might be accidentally detected in clinical practice, GS should be suspected if three or more osteomas are found.^2^ Treatment is not indicated for the osteoma; however, surgery might be required for mandibular dyskinesia or cosmesis.^2^

If left untreated, patients with FAP will develop colorectal cancer,^4^ which is the leading cause of death in this population.^5^ Prophylactic colectomy is a reliable treatment; however, surgery is recommended for patients in their twenties. In patients with GS, the mutation in *APC*, which is a tumor‐suppressor gene, might lead to complications such as gastric cancer, duodenal cancer, thyroid cancer, and malignant dental tumors, in addition to colon cancer.^4^, ^5^, ^6^ The presence of desmoid tumors also greatly influences the prognosis.^4^, ^5^ Therefore, full‐body examination and long‐term follow‐up are required.

An otolaryngologist might come across a patient of GS presenting with nasal and buccal symptoms such as sinusitis and dental infection. GS should be suspected if the characteristic findings—including multiple osteomas of the skull and facial bones, excess teeth, impacted teeth, and odontomas—are observed. Eliciting a family history and whole‐body examination, including the colon, can contribute to early diagnosis and treatment, potentially improving the patient's prognosis.

## 4 CONCLUSION

Gardner syndrome with odontogenic sinusitis is rare; however, it should be suspected in patients with multiple osteomas of the skull and facial bones, excess teeth, impacted teeth, and odontomas. Early diagnosis and treatment of GS might improve the prognosis. Further case studies are necessary to determine the optimal strategy for treating patients with GS and odontogenic sinusitis.

## CONFLICT OF INTEREST

The authors have no funding, financial relationships, or conflicts of interest to declare.

## AUTHOR CONTRIBUTION

All authors revised the manuscript, approved the manuscript to be published, and agree to be accountable for all aspects of the work in ensuring that questions related to the accuracy or integrity of the work are appropriately investigated and resolved.

## STATEMENT OF INSTITUTIONAL REVIEW BOARD APPROVAL

Ethical approval for this case report was obtained from the institutional review board of Tokai University Hospital (approval number 20R‐352). The study was carried out in accordance with the Code of Ethics of the World Medical Association (Helsinki Declaration). The institutional review board has owned the responsibility for the anonymization of the patient, and the requirement for informed consent was waived.

## Data Availability

The data that support the findings of this study are openly available at http://doi.org/10.1002/ccr3.4256.

## References

[1] Gardner EJ , Richards RC . Multiple cutaneous and subcutaneous lesions occurring simultaneously with hereditary polyposis and osteomatosis. Am J Hum Genet. 1953;5(2):139‐147. 13065261 PMC1716470

[2] de Oliviera RM , Martins WD , de Sousa MH , et al. Oral and maxillofacial manifestations of familial adenomatous polyposis (Gardner's syndrome): a report of two cases. J Contemp Dent Pract. 2009;10(1):82‐90. 19142260

[3] Shrotriya A , Chaurasia A , Sharma P , Kumari N , Safi S , Rastogi S . Odontomas: an unusual case series associated with infection and cutaneous fistula formation. Dentistry. 2018;8:9.

[4] Jasperson KW , Patel SG , Ahnen DJ . APC‐associated polyposis conditions. 1998 Dec 18 [Updated 2017 Feb 2]. In: Adam MP , Ardinger HH , Pagon RA , et al., eds. GeneReviews® [Internet]. Seattle, WA: University of Washington; 1993‐2021. https://www.ncbi.nlm.nih.gov/books/NBK1345/. Accessed March 15, 2021.

[5] Iwama T , Tamura K , Morita T , et al. A clinical overview of familial adenomatous polyposis derived from the database of the polyposis registry of Japan. Int J Clin Oncol. 2004;9(4):308‐316. 15375708 10.1007/s10147-004-0414-4

[6] Fitzpatrick SG , Hirsch SA , Listinsky CM , Lyu DJ‐H , Baur DA . Ameloblastic carcinoma with features of ghost cell odontogenic carcinoma in a patient with suspected Gardner syndrome. Oral Surg Oral Med Oral Pathol Oral Radiol. 2015;119(4):e241‐245. 10.1016/j.oooo.2014.09.028 25434693

